# Cortical Networks of Creative Ability Trace Gene Expression Profiles of Synaptic Plasticity in the Human Brain

**DOI:** 10.3389/fnhum.2021.694274

**Published:** 2021-07-26

**Authors:** William Orwig, Ibai Diez, Elisenda Bueichekú, Patrizia Vannini, Roger Beaty, Jorge Sepulcre

**Affiliations:** ^1^Department of Radiology, Gordon Center for Medical Imaging, Massachusetts General Hospital and Harvard Medical School, Boston, MA, United States; ^2^Athinoula A. Martinos Center for Biomedical Imaging, Massachusetts General Hospital and Harvard Medical School, Charlestown, MA, United States; ^3^Department of Neurology, Brigham and Women’s Hospital, Boston, MA, United States; ^4^Department of Neurology, Massachusetts General Hospital, Boston, MA, United States; ^5^Department of Psychology, The Pennsylvania State University, University Park, PA, United States

**Keywords:** creativity, fMRI, functional connectivity, genetics, synaptic plasticity

## Abstract

The ability to produce novel ideas is central to societal progress and innovation; however, little is known about the biological basis of creativity. Here, we investigate the organization of brain networks that support creativity by combining functional neuroimaging data with gene expression information. Given the multifaceted nature of creative thinking, we hypothesized that distributed connectivity would not only be related to individual differences in creative ability, but also delineate the cortical distributions of genes involved in synaptic plasticity. We defined neuroimaging phenotypes using a graph theory approach that detects local and distributed network circuits, then characterized the spatial associations between functional connectivity and cortical gene expression distributions. Our findings reveal strong spatial correlations between connectivity maps and sets of genes devoted to synaptic assembly and signaling. This connectomic-transcriptome approach thus identifies gene expression profiles associated with high creative ability, linking cognitive flexibility to neural plasticity in the human brain.

## Introduction

Creativity represents a defining quality of human cognition. Given the complexity of human creativity, the production of novel ideas cannot be attributed to a single region of the brain. Rather, increasing neuroscientific evidence links creative thinking to a complex interplay of interconnected brain networks (Beaty et al., 2019). However, a detailed characterization of the brain networks and neurobiological assembly supporting creativity remains elusive. Previous work using functional brain imaging has identified cortical networks involved in idea production (Beaty et al., 2016, 2018), with a majority of neuroscientific work focusing on divergent thinking–the ability to produce original ideas in response to open-ended problems (Plucker and Makel, 2010). Task-based fMRI studies of divergent creative thinking have revealed dynamic interactions between cognitive brain networks (Beaty et al., 2015; Li et al., 2017), and resting-state fMRI studies have further characterized intrinsic connectivity networks associated with creative ability (Takeuchi et al., 2012; Beaty et al., 2014; Shi et al., 2018). To date, however, the biological significance of such large brain networks supporting creative thinking remains unknown, due in part to the challenge of linking distributed connectivity networks to underlying neuronal properties, such as synaptic signaling and plasticity. Here, we sought to overcome these limitations by applying a novel connectomic-transcriptome approach to identify the spatial intersection between brain connectivity phenotypes related to creative ability and cortical genetic expression profiles, providing insight into the neurobiology of creativity.

Given the biological complexity of the human brain–a structure that, at the macroscale level, is organized as a system of interconnected networks–there is a growing demand for analytical strategies that capture such complexity. Network neuroscience and graph theory approaches can help to address this problem (Fornito et al., 2016; Bassett and Sporns, 2017). Graph theory analyses improve our ability to describe functional brain networks and study the segregation and integration patterns of connectivity. Conceptual models of the human brain suggest a hierarchical network architecture, starting in primary unimodal cortices and progressing toward areas of multimodal integration (Sepulcre, 2014). Research into the functional and structural composition of network assembly has revealed modules with dense local connectivity associated with specific cognitive function, and cortical hubs which integrate information between distributed modules (Bassett and Bullmore, 2006; Bullmore and Sporns, 2009; Sepulcre et al., 2012). Areas of multimodal integration, displaying distributed connectivity across networks, are particularly important for higher-order cognitive function, like creativity.

With the advent of new methods combining human neuroimaging data with whole-brain cortical gene expression (Diez and Sepulcre, 2018; Xin et al., 2019; Bueichekú et al., 2020), it is now possible to characterize neurobiological features of complex human cognitive abilities, such as creativity. Here, we leverage a novel connectomic-transcriptome methodology to provide insight into the network neurobiology of individual creative ability. Using computational models of semantic distance, we objectively quantify the creative quality of ideas generated by a large sample of participants (*n* = 175) on a common psychometric assessment of divergent creative thinking (i.e., the alternate uses task). We define neuroimaging phenotypes associated with creative ability using a graph theory approach applied to resting-state fMRI data that detects local and distributed functional connectivity (Sepulcre et al., 2012), then characterize the spatial associations between connectivity patterns and cortical gene expression distributions using the Allen Human Brain Atlas (AHBA). We hypothesized that distributed connectivity networks would be related to individual differences in creative ability. Additionally, we hypothesized that cortical expression of genes involved in synaptic plasticity would share high spatial similarity with the distributed connectivity maps. We thus provide a neurogenetic profile of highly creative individuals and identify cortical expression of specific genes related to high creative ability.

## Materials and Methods

### Participants

The total sample consisted of 175 participants (127 women, mean age = 22.67 years, SD = 6.37). All participants were right-handed with normal or corrected-to-normal vision and reported no history of any neurological disorders, cognitive disabilities, or medications that affect the central nervous system (Beaty et al., 2018). All participants provided written informed consent and the study was approved by the UNCG Institutional Review Board.

### Creativity Assessment

Divergent thinking (DT) performance was assessed by the Alternative Uses Task (AUT), conducted during a separate task-based fMRI scan (Beaty et al., 2018), as well as on a computer outside the scanner. Note that the task-based fMRI data are not presented here (only the verbal responses; Beaty et al., 2018). During the task-based fMRI scan, participants were presented with a series of everyday objects (e.g., brick) and asked to imagine new and unusual uses for each object. Participants had 12 s to think of a single alternate use for a list of 23 objects, then had 5 s to verbally report their response via an MRI compatible microphone (Beaty et al., 2018; Benedek et al., 2019). For the computer-based assessment, participants had 3 min to generate as many alternative uses for two objects as possible (box and rope).

To objectively quantify the creative quality of responses, we used several computational models of semantic distance (Beaty and Johnson, 2020). Semantic distance captures the novelty facet of creativity by computing the cosine similarity of concepts in large corpora of natural language (Prabhakaran et al., 2014; Kenett, 2019; Beaty and Johnson, 2020). We computed semantic distance using an online application called *SemDis*, an open platform developed to automate creativity assessment via semantic distance^1^ (Beaty and Johnson, 2020). *SemDis* leverages five compositional vector models to compute the relatedness between inputted texts: three continuous bag of words (CBOW) predict models and two count models. CBOW/predict models were built using a neural network architecture (Mandera et al., 2017) that employs a sliding window to move through text corpora and aims to predict a central word from surrounding context words (cf., *word2vec*); count models, in contrast to predict models, compute the co-occurrence of words within these large text corpora. All five spaces were used to compute the semantic distance between the AUT item (e.g., box) and participants’ responses, where the cosine angle between the word vectors represents semantic similarity; semantic distance is then computed by subtracting this semantic similarity score from one (Prabhakaran et al., 2014; Green, 2016; Beaty et al., 2017; Kenett and Faust, 2019). Following Beaty and Johnson (2020), we used latent variable modeling to extract the common variance from the five semantic models. This approach has the benefit of reducing the influence of any one model–which has been shown to yield idiosyncratic values (Mandera et al., 2017) specific to the given model and text corpus employed, thus boosting the reliability and generalizability of results.

## Functional MRI Acquisition and Image Processing

Resting-state fMRI data were acquired for all participants on a 3T Siemens Magnetom MRI system using a 16-channel head coil. High resolution T1 scans were acquired for anatomical normalization. Blood-oxygenation-level-dependent (BOLD) T2^∗^-weighted functional images were acquired with gradient echo-planar imaging sequence with the following parameters: TR = 2,000 ms, TE = 30 ms, flip angle = 78°, 192 mm FoV, 32 axial slices, 3.5 × 3.5 × 4.0 mm, interleaved slice ordering, sequence length = 5 min. Participants were instructed to relax awake in the scanner with eyes closed for the duration of the scan. MRI data for both anatomical and functional images were preprocessed using FMRIB Software Library v5.0.7 (FSL) and MATLAB 2017a (Mathworks Inc., Natick, MA, United States). The anatomical and functional preprocessing pipelines were adapted from previous work (Diez et al., 2019). The anatomical T1 preprocessing included: reorientation to right-posterior-inferior; alignment to anterior and posterior commissures; skull stripping; gray matter, white matter and cerebrospinal fluid segmentation; and computation of non-linear transformation between individual skull-stripped T1 and 2 mm resolution MNI152 template images. The functional MRI preprocessing pipeline included: slice time correction; reorientation to RPI; realigning functional volumes within runs with a rigid body transformations (six parameters linear transformation); computation of the transformation between individual skull-stripped T1 and mean functional images; intensity normalization; removal of confounding factors from the data using linear regression–including 12 motion-related covariates (rigid motion parameters and its derivatives), linear and quadratic terms, and five components each from the lateral ventricles and white matter. Global signal regression was not applied due to the spurious correlations this can introduce. Transformation of resting-state data to MNI space was performed, concatenating the transformation from functional to structural and from structural to MNI, spatial smoothing with an isotropic Gaussian kernel of 6-mm FWHM, and band-pass filtering (0.01–0.08 Hz) to reduce low-frequency drift and high-frequency noise were also applied. Head motion was quantified using realignment parameters obtained during image preprocessing, including three translation and three rotation estimates. Scrubbing of time points with excess head motion interpolated all time points with a frame displacement > 0.2 mm was applied. While several participants demonstrated head motion above this 0.2 mm threshold, we correct for this motion by interpolating time points before and after. The distributions of the correlations across time series were reviewed for possible contamination; no outliers were observed from the whole-brain connectivity distributions.

## Local and Distributed Connectivity

**FIGURE 1:**
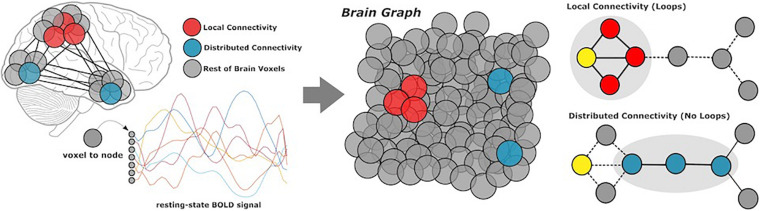
Methods overview. Participants underwent resting-state functional MRI scans, from which we extracted BOLD data of all gray matter voxels in the cerebral cortex. Based on coactivation patterns between voxels, we performed local and distributed functional connectivity analysis to characterize network modularity. Local connectivity describes links within modules, whereas distributed connectivity captures the relatively smaller set of links which communicate between modules.

Complex brain networks are highly modular, meaning that nodes (or voxels) are organized into local communities, corresponding to specific cognitive functions. Local connectivity describes the large number of connections within well-defined communities; distributed connectivity represents the relatively small set of links which communicate across modules (Figure 1). We generated local and distributed maps using whole-brain stepwise functional connectivity analyses (Sepulcre et al., 2012). Additionally, we conducted weighted degree (WD) analysis to calculate all links in the brain, then identify individual variability between WD maps for local and distributed connectivity (Diez and Sepulcre, 2018). Local and distributed maps were computed in different regression analyses.

To compute the voxel-wise stepwise connectivity maps, we followed the steps described below.

$$\begin{array}{c} {\text{NSFC}}_{1}\,\left(i,j\right)=\frac{r\,\left(i,j\right)-\text{min}\,\left(r\right)}{\max\left(r\right)-\text{min}\,\left(r\right)}\\ {\text{SFC}}_{s}\left(i,j\right)=\sum_{k=1}^{n}{\text{NSFC}}_{s-1}\left(i,k\right){\text{NSFC}}_{1}\left(k,j\right)\left[i\neq j,s>1\right]\\ {\text{NSFC}}_{s}=\frac{{\text{SFC}}_{s}-\text{min}\,\left({\text{SFC}}_{s}\right)}{\text{max}\,\left({\text{SFC}}_{s}\right)-\text{min}\,\left({\text{SFC}}_{s}\right)} \end{array}$$

Where *r* is the fdr corrected association connectivity matrix, *i* and *j* represents a pair of voxels, *n* is the number of voxels, and NSFC*_s_* is the normalized stepwise connectivity matrix for number of steps (*s*).

The local connectivity matrices were computed as:

$$\begin{array}{c} {\text{NLC}}_{1}\left(i,j\right)=\frac{r\,\left(i,j\right)-\text{min}\,\left(r\right)}{\max\left(r\right)-\text{min}\,\left(r\right)}\left[{\text{NSF}}_{2}\left(i,j\right)\neq 0\right]\\ {\text{LC}}_{s}\left(i,j\right)=\sum_{k=1}^{n}{\text{NLC}}_{s-1}\left(i,k\right){\text{NLC}}_{1}\left(k,j\right)\left[i\neq j,s>1\right]\\ {\text{NLC}}_{s}=\frac{{\text{LC}}_{s}-\text{min}\,\left({\text{LC}}_{s}\right)}{\text{max}\,\left({\text{LC}}_{s}\right)-\text{min}\,\left({\text{LC}}_{s}\right)} \end{array}$$

The distributed connectivity matrices were computed as:

$$\begin{array}{c} {\text{NDC}}_{1}\left(i,j\right)={\text{NSFC}}_{4}\left(i,j\right)\left[{\text{NSFC}}_{1}\left(i,j\right)={\text{NSFC}}_{2}\left(i,j\right)=0\right]\\ {\text{DC}}_{s}\left(i,j\right)=\sum_{k=1}^{n}{\text{NDC}}_{s-1}\left(i,k\right){\text{NSFC}}_{1}\left(k,j\right)\left[i\neq j,s>1\right]\\ {\text{NDC}}_{s}=\frac{{\text{DC}}_{s}-\text{min}\,\left({\text{DC}}_{s}\right)}{\text{max}\,\left({\text{DC}}_{s}\right)-\text{min}\,\left({\text{DC}}_{s}\right)} \end{array}$$

Final WD maps for local and distributed connectivity were computed as the sum of steps 2–7.

After obtaining local and distributed maps for all participants, a general linear model was used to compute the association between connectivity and DT. For each voxel, a statistical analysis was applied. DT was used as the independent variable and that particular voxel value (local or distributed connectivity) was the dependent variable. All statistical analyses were corrected for participant age and sex. Whole-brain correction for multiple comparisons was computed using Monte Carlo simulation with 10,000 iterations to estimate the probability of positive clusters with a two-tailed *p*-value < 0.05 (3dClustSim^2^).

To reduce the dimensionality of the functional networks, after preprocessing the time series were down-sampled to 6 mm. Pearson Correlation was used to obtain a 5,742 × 5,742 voxel-wise connectivity matrix. Then, Fisher transformation was applied, and negative values were removed due to their controversial interpretation in graph theory integration analysis (Qian et al., 2018). A false discovery rate (fdr) correction of *q*-level = 0.005 was applied to remove the weakest connections.

## Combination of Neuroimaging and Cortical Gene Expression

### Spatial Similarity Analysis

We used the AHBA to study the spatial similarity between protein-coding genetic profiles and our local-distributed connectivity maps associated with DT. The AHBA provides whole-brain expression distributions for six human subjects (Hawrylycz et al., 2012). The atlas is comprised of 20,737 protein-coding genes, based on 58,692 measurements of gene expression in 3,702 brain samples taken from these six subjects. One limitation of this approach is that the AHBA subjects are mostly male, whereas the fMRI data was collected in a sample containing mostly females. We used anatomical surface transformations of these genetic transcription profiles within 68 cortical regions of the Desikan–Killiany atlas (Desikan et al., 2006) to capture mean cortical expression of genes across these regions. Spatial Similarity Analysis was conducted by means of a MATLAB in-house coding, available upon request (MATLAB R2015b; The MathWorks INC.). The objective of this analysis was to identify which genes, from the 20,737 genes of the AHBA, had a cortical expression profile corresponding to the connectivity maps associated with DT. We created a null hypothesis distribution, comparing the entire AHBA transcriptome with the DT network connectivity map, then generated a list of candidate genes with cortical expression profiles associated with DT. We identify genes with both highly positive and negative scores, beyond a significance threshold (>2 SD).

## Biological Processes of Genes Mediating DT

The list of genes whose spatial cortical expression demonstrated inverse spatial correlation with the local and distributed DT connectivity network was entered in a GO term enrichment analysis tool (The Gene and Ontology Consortium, 2019). GO is an open-access, genetic annotation resource available to investigate gene functionalities. To characterize our findings within GO, we used the annotation system of biological processes. Biological processes deal with gene function that lead to specific objectives, often in a highly regulated manner and particular temporal sequence (The Gene and Ontology Consortium, 2019). The Protein Analysis Through Evolutionary Relationships (PANTHER) resources enable inference about gene functions, by classifying protein sequences in terms of their evolutionary history and function. We ran a PANTHER Overrepresentation Test (Mi et al., 2017) using the combined lists of genes whose cortical expression demonstrated high spatial correlations with local and distributed connectivity maps associated with DT. A Bonferroni correction for multiple comparison was run at threshold *q* < 0.05. The results of the PANTHER analysis were based on the relative term enrichment, which indicates the degree to which each gene is represented in a given set of genes.

## Interactome Analysis

Using an interactome approach, we validated our genetic results beyond the spatial similarities with the local and distributed DT cortical maps. We used Cytoscape (Lopes et al., 2010) and Genemania (Mostafavi et al., 2008) to perform an interactome analysis and closeness centrality assessment of the set of candidate genes obtained from the spatial similarity analysis. We used the Genemania composite gene–gene interaction profile from predicted physical interactions and shared protein domains to determine centrality of the identified genes within the combined genetic functional network.

## Results

### Local and Distributed Connectivity

**FIGURE 2:**
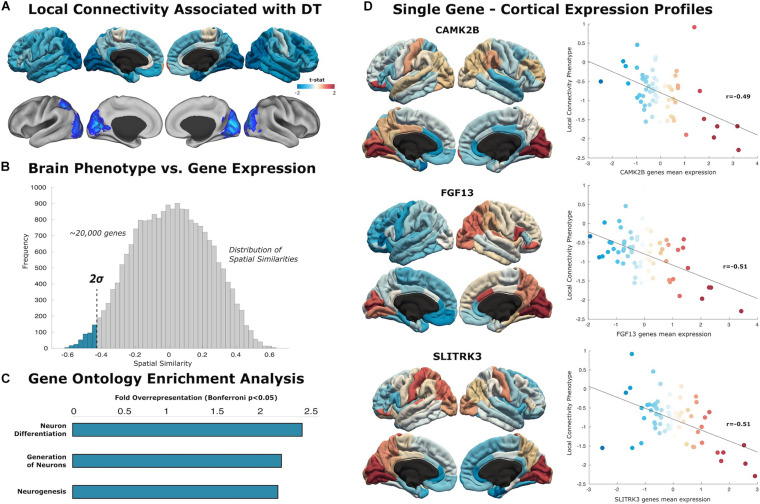
Local connectivity maps. **(A)** Cortical surfaces display t-statistic of the regression between the local connectivity and DT. Above, data is projected to 68 cortical regions of the Desikan–Killiany atlas to capture mean connectivity across these regions. Below, projection of the t-statistic at the voxel-level, showing regions that survive to multiple comparison with cluster wise Monte Carlo simulation. Each analysis result is displayed in left and right hemispheric surfaces, with lateral and medial projections. **(B)** The similarity distribution represents the results of topographical similarity analysis between the local connectivity network and the brain transcriptome maps (∼20,000 protein-coding genes). Genes which have a highly negative similarity score (below dotted line at 2 SDs) are inversely related to our local connectivity map, meaning they are highly expressed in regions with low connectivity. **(C)** Gene Ontology enrichment analysis reveals this subset of 363 genes exhibit overrepresented functionalities in key domains of neuron generation and differentiation (FE > 2; statistically significant FDR-corrected *q* < 0.05). **(D)** Scatterplots show the spatial similarity relationship throughout brain regions between independent expression of three genes (CAMK2B, FGF13, and SLITRK3) and the local connectivity map (linear fit represented with a black line).

**FIGURE 3:**
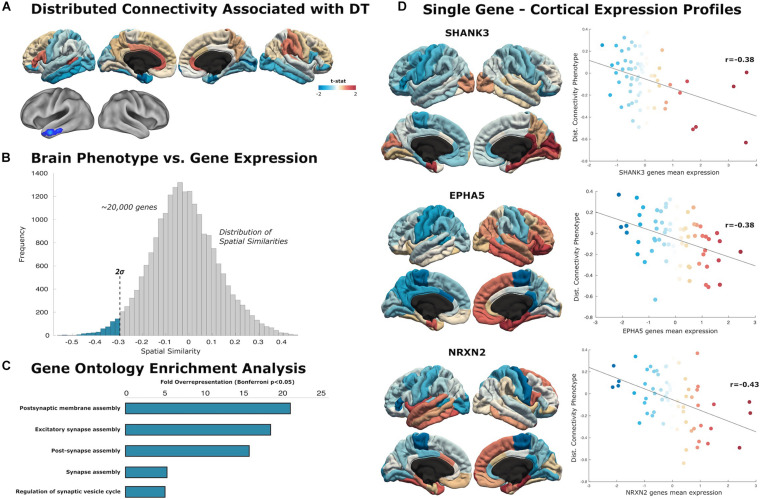
Distributed connectivity maps. **(A)** Cortical surfaces display *t*-statistic of the regression between the distributed connectivity and DT. Above, data is projected to 68 cortical regions of the Desikan–Killiany atlas to capture mean connectivity across these regions. Below, cortical projection of the t-statistic at the voxel-level, showing regions that survive to multiple comparison with cluster wise Monte Carlo simulation. **(B)** The similarity distribution represents the results of topographical similarity analysis between the distributed connectivity network and the brain transcriptome maps (∼20,000 protein-coding genes). Genes which have a highly negative similarity score (below dotted line at 2 SDs) are inversely related to our distributed connectivity map, meaning they are highly expressed in regions with low connectivity. **(C)** Gene Ontology enrichment analysis reveals this subset of 516 genes exhibit overrepresented functionalities in key domains of post-synaptic assembly (FE > 15; statistically significant FDR-corrected *q* < 0.05). **(D)** Scatterplots show the spatial similarity relationship throughout brain regions between independent expression of three genes (SHANK3, EPHA5, and NRXN2) and the distributed connectivity map (linear fit represented with a black line).

We generated separate local and distributed maps using whole-brain stepwise functional connectivity analyses applied to resting-state fMRI data (Sepulcre et al., 2012). Additionally, we conducted weighted degree (WD) analysis to calculate all links in the brain, then identify individual variability between WD maps for local and distributed connectivity (Diez and Sepulcre, 2018). Local connectivity maps revealed a negative association between individual creative ability and connectivity within the primary occipital cortex, lateral occipital cortex, and inferior parietal areas (Figure 2). Distributed connectivity from the left inferior temporal lobe was negatively associated with creative ability (Figure 3). Thus, the ability to produce novel ideas–quantified objectively using computational semantic distance–was negatively associated with local connectivity within occipital-parietal regions and distributed connectivity to the left temporal pole.

## Genes With Associated Cortical Expression Profiles

To characterize the neurobiological basis of creative ability, we compared local and distributed connectivity maps with the cortical expression of genes (*N* = 20,737) from the AHBA. We obtained a similarity score between each comparison, then identified genes in the lower tail which were highly expressed in cortical regions with lower connectivity. Resulting lists contained 363 and 516 genes whose cortical expression levels were associated with local and distributed connectivity maps, respectively (Supplementary Table 1). Among these, we find 125 shared genes, present in both local and distributed lists (Supplementary Table 1). We then performed a PANTHER overrepresentation analysis of these gene lists to identify significant roles in specific biological processes [Fisher’s Exact, Bonferroni correction for multiple comparison and fold enrichment (FE) > 2].

### Local Genes

Similarity analysis revealed spatial correlations between our local connectivity profiles and cortical expression of genes involved in neuron generation and differentiation (Figure 2). The set of genes associated with local connectivity maps exhibited overrepresented functionalities in key domains of neuron differentiation (FE = 2.40), generation of neurons (FE = 2.16), and neurogenesis (FE = 2.13). Among these genes, we highlight several with high spatial correlation to the local connectivity phenotype: **CAMK2B** (*r* = −0.49, *p* < 0.001), **FGF13** (*r* = −0.51, *p* < 0.001), **SLITRK3** (*r* = 0.51, *p* < 0.001).

### Distributed Genes

**FIGURE 4:**
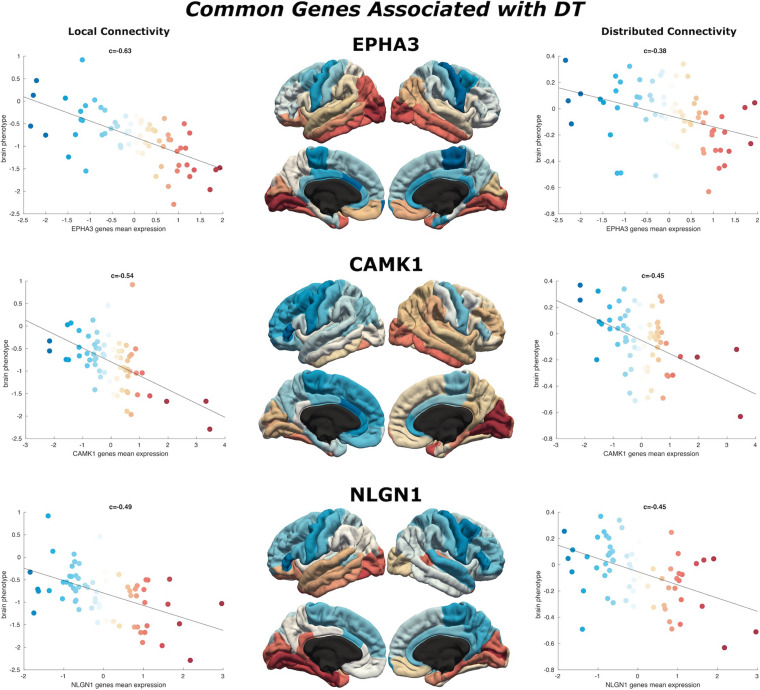
Common genes associated with DT. Scatterplots show the spatial similarity relationship throughout brain regions between independent expression of three genes (EPHA3, CAMK1, and NLGN1), the local connectivity map (*left*) and the distributed connectivity map (*right*). Linear fit represented with a black line.

We identified a list of candidate genes that share a spatial correlation with our distributed connectivity phenotype (Figure 4). The genes associated with distributed connectivity were more functionally engaged in postsynaptic membrane assembly (FE = 20.93), excitatory synapse assembly (FE = 17.94), post-synapse assembly (FE = 15.43), synapse assembly (FE = 5.28), and regulation of synaptic vesicle cycle (FE = 4.97). From the nineteen total genes identified in the GO database as being associated with post-synapse assembly, our distributed gene list contained seven. We highlight individual genes sharing high spatial similarity with the distributed connectivity maps: **SHANK3** (*r* = −0.38, *p* < 0.001), **EPHA5** (*r* = −0.38, *p* < 0.001), **NLGN2** (*r* = −0.32, *p* < 0.01), **NRXN1** (−0.28, *p* < 0.05), and **NRXN2** (*r* = −0.43, *p* < 0.001).

### Convergence of Local and Distributed Genes

**FIGURE 5:**
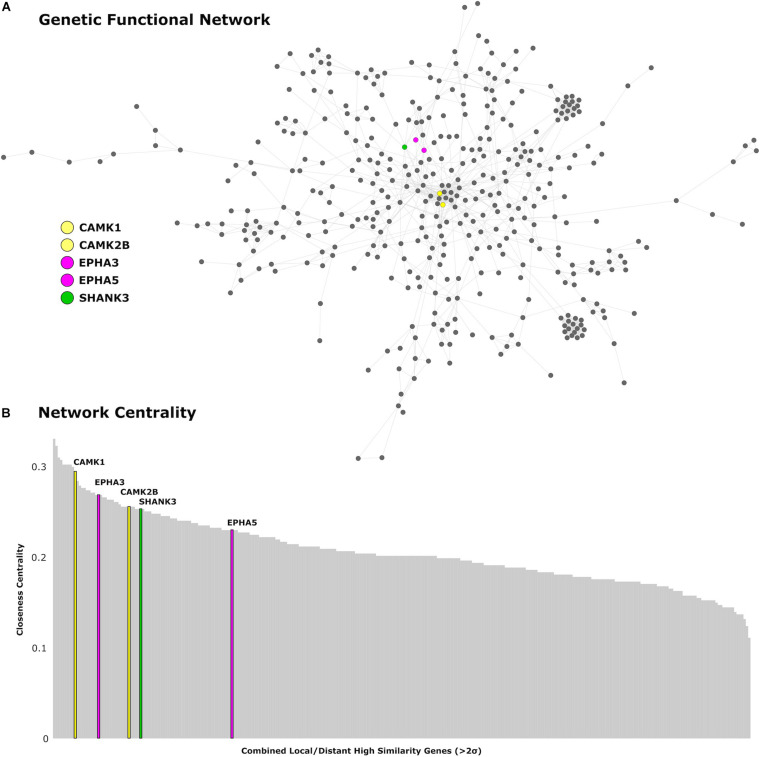
Genetic network analysis. **(A)** Protein-coding genes from the AHBA with high cortical expression within the local and distributed networks (>2 SDs) are presented in the network topological space. **(B)** Genes are plotted as a function of the closeness centrality in the network. Genes related to synaptic plasticity and post-synaptic assembly are highlighted in corresponding colors. CAMK1 and CAMK2B genes are represented in yellow, EPHA3 and EPHA5 are represented in magenta, and SHANK3 is represented in green.

We find 125 genes present in both local and distributed lists (Figure 4). Among these, we identify **CAMK1** to be significantly associated with local (*r* = −0.54, *p* < 0.001) and distributed (*r* = −0.45, *p* < 0.001) connectivity maps. **NLGN1** also shared a spatial correlation with both local (*r* = −0.49, *p* < 0.001) and distributed (*r* = −0.45, *p* < 0.001) maps. In addition, **EPHA3** shared high spatial correlation with local maps (*r* = −0.63, *p* < 0.001) and moderate association with distributed maps (*r* = −0.38, *p* < 0.001). Similarly, **NPTN** shared a highly significant correlation with local maps (*r* = −0.64, *p* < 0.001) and moderate association with distributed maps (*r* = −0.37, *p* < 0.05). Moreover, we found that cortical expression of two GABAergic protein coding genes–GABRA6 (*r* = −0.52, *p* < 0.001) and GABRB2 (*r* = −0.53, *p* < 0.001)–shared high spatial similarity with the local connectivity map. These genes were also present in the distributed gene lists, though correlations were not as strong–GABRA6 (*r* = −0.30, *p* < 0.05) and GABRB2 (*r* = −0.27, *p* < 0.05). An interactome-based validation approach, with independent gene-gene interaction profiles, revealed that CAMK1, CAMK2B, EPHA3, EPHA5, and SHANK3 were centrally localized within the gene interaction network of combined (local and distributed) lists of genes (Figure 5).

## Discussion

Despite the critical importance of creativity, the neurobiology of human creativity has remained undefined. For the first time, we provide a comprehensive gene-brain model of high creative ability, combining computational modeling of behavior with a novel connectomic-transcriptome analysis to identify a neurobiological profile characterizing high creative ability. We identified brain connectivity phenotypes through the application of novel graph theory metrics of local and distributed connectivity, revealing patterns of functional network organization related to creative ability. Moreover, AHBA data of gene expression levels permitted us to characterize topological distributions of protein coding genes in relation to functional connectivity profiles. Critically, we found that the spatial topology of genes devoted to the regulation of postsynaptic assembly and synapse organization resembled the distributed connectivity related to high creative ability, thus linking cognitive flexibility with neural plasticity in the highly creative brain. We discuss our findings in relation to specific genes previously associated with synapse formation and plasticity.

## Post-synaptic Organization Modulates Distributed Connectivity

We found that distributed connectivity profiles shared a high correlation with the spatial cortical expression of genes involved in postsynaptic assembly and synapse organization. Among these genes, we identify SHANK3, NRXN1, NRXN2, NLGN1, and NLGN2 to share spatial topology with the distributed connectivity map. Neurexins (NRXN) and neuroligins (NLGN) are synaptic cell adhesion molecules; NRXN are primarily located in presynaptic membrane and NLGN are present in postsynaptic membrane (Bang and Owczarek, 2013). The SHANK family of protein coding genes, located in the post-synaptic density of glutamatergic synapses, has been associated with synapse assembly, postsynaptic density assembly, and regulation of long-term synaptic potentiation. Our results indicate higher expression of SHANK3 in association with decreased functional connectivity in the left temporal pole, an area known to be involved in associative memory and semantic processing. Higher expression of SHANK3 in more creative individuals may promote efficient synaptic organization within this region, reflected in lower functional connectivity.

The NLGN–NRXN–SHANK pathway plays a primary role in regulating synaptic formation, maturation, and plasticity. NRXN and NLGN play a central role in presynaptic and postsynaptic differentiation (Craig and Kang, 2007). Additionally, the neuroligin-neurexin complex is thought to promote maturation and organization of synapses through bidirectional signaling (Taniguchi et al., 2007). Studies of NRXN and NLGN knockout in mice indicate an essential role in synapse maturation and function (Südhof, 2008). Our findings suggest that cortical expression of genes involved in the assembly of synapses in the human brain are essential for shaping the neural networks which underpin creativity. Specifically, the biological processes of post-synaptic organization are overrepresented in our set of genes associated with the creative ability in the brain.

### Bidirectional Synaptic Plasticity in the Creative Brain

Investigation of general genotype-phenotype connectivity maps highlighted select genes linked to nervous system development and synaptic plasticity. CAMK1 and CAMK2B are members of calcium/calmodulin-dependent protein kinase, involved in synapse formation and neuronal plasticity. CAMK2B is a protein-coding gene which plays an essential role in synaptic plasticity and maturation, promoting synapse formation, particularly in hippocampal neurons. CAMK2B expression promotes synaptic remodeling in mature neurons. Previous work has shown an influx in CAMK2 activity in post synaptic density of dendrites following LTP induction (Strack et al., 1997). We find a high spatial similarity between CAMK2B and our local connectivity maps. This shared topology possibly indicates a role of CAMK2B in synaptic organization which enables creative thinking. Defining the genetic basis of bidirectional synaptic plasticity may be essential for understanding the flexible cognitive processes involved in creative problem solving.

Related to synaptic plasticity, we find two prominent genes belonging to the ephrin (EPH) family of cell adhesion proteins. A distinctive feature of these binding proteins is in the bidirectional communication between neurons (Pasquale, 2008). EPHA3 is involved in axon guidance and synaptic plasticity. EPHA3 shared a strong negative spatial correlation with our local connectivity phenotype–higher expression was associated with lower connectivity. Regulation of bidirectional communication is also thought to depend on the interaction between presynaptic NRXN and postsynaptic NLGN, described above. Among our set of genes associated with our local and distributed connectivity phenotypes, we identify an overrepresented sample of genes involved in bidirectional synapse communication. This finding suggests bidirectional communication, reflected in lower functional connectivity at rest, may be central to the neurobiological organization of creativity in the brain.

Neuroplastin (NPTN) is a protein-coding gene involved in synaptic plasticity. GO database specifies NPTN expression in relation to axon guidance and dendrite self-avoidance–the process by which dendrites avoid contact with sister dendrites of the same cell. Extensive study in mice has demonstrated retrograde amnesia after an associative learning task induced by NPTN ablation (Bhattacharya et al., 2017). Our results show NPTN as sharing a spatial correlation with both local and distributed connectivity maps: increased NPTN expression was related to lower functional connectivity. We interpret this negative association as a neurobiological profile, wherein cortical expression of these select neuroplasticity genes underlies individual differences in brain functional organization related to creativity.

### Inhibitory Neurotransmission in Creative Brain Networks

Gamma-Aminobutyric acid (GABA) is the predominant inhibitory neurotransmitter in the human brain and mediates synaptic inhibition as a GABA-gated ion channel. Among our lists of candidate genes, both NRXN1 and NLGN2 are associated with inhibitory synapse formation. Additionally, we identified two protein coding genes involved in GABAergic synaptic transmission: GABRB2 and GABRA6. These genes play a role in inhibitory synapse assembly and regulation of postsynaptic membrane potential (The Gene and Ontology Consortium, 2017). Studies of resting-state GABA concentrations have revealed high regional GABA expression within Default Mode Network regions is associated with enhanced deactivation (Hu et al., 2013). Recent investigation of the genetic basis of creativity has suggested GABA/Glutamate ratio may be closely related to creative ability (Liu et al., 2018). Transcutaneous vagus nerve stimulation (tVNS) further supports the role of GABA in creative thinking (Colzato et al., 2018). Our findings indicate that cortical expression of GABAergic genes is related to regional functional connectivity in the creative brain at rest. We thus find a negative association between expression of inhibitory neurotransmitters and functional connectivity. The present study further highlights the role of inhibitory neurotransmission in creativity. Genes modulating synaptic inhibition may be essential to understanding the neural circuitry underpinning creativity, but more work is needed to explain how these biological processes of inhibitory neurotransmission facilitate creative cognition.

## Conculsion

Our results identified neurogenetic underpinnings of the cortical connectivity of creativity, highlighting their contribution to synapse plasticity. More work is needed to understand the role of synaptic plasticity in creative cognition. Future research could explore possible manipulation of cortical plasticity via non-invasive brain stimulation, to selectively induce or inhibit functional communication between large-scale networks. Taken together, our results pave the way toward elucidating the complexity of brain networks and neurobiological mechanisms underlying human creativity.

## Data Availability Statement

The raw data supporting the conclusions of this article will be made available by the authors, without undue reservation.

## Ethics Statement

The studies involving human participants were reviewed and approved by University of North Carolina, Greensboro Institutional Review Board. The patients/participants provided their written informed consent to participate in this study.

## Author Contributions

WO conducted the analyses and wrote the manuscript. ID developed the methods and helped in the preprocessing of the imaging data. EB assisted with revisions of the manuscript and contributed conceptually to the project. RB collected the data and helped revise the manuscript. PV helped revise the manuscript. JS assisted with the conceptualization of the project and revised the manuscript. All authors contributed to the article and approved the submitted version.

## Conflict of Interest

The authors declare that the research was conducted in the absence of any commercial or financial relationships that could be construed as a potential conflict of interest.

## Publisher’s Note

All claims expressed in this article are solely those of the authors and do not necessarily represent those of their affiliated organizations, or those of the publisher, the editors and the reviewers. Any product that may be evaluated in this article, or claim that may be made by its manufacturer, is not guaranteed or endorsed by the publisher.

## Footnotes

1 http://semdis.wlu.psu.edu

2 http://afni.nimh.nih.gov
