# Supplementary material for: Cortical Networks of Creative Ability Trace Gene Expression Profiles of Synaptic Plasticity in the Human Brain
Source: Front Hum Neurosci. 2021 Jul 26;15:694274. doi: 10.3389/fnhum.2021.694274 (PMC8350487; doi:10.3389/fnhum.2021.694274)
Supplement: Supplementary file 1 [file Data_Sheet_1.PDF]

Supplementary Table 1. Gene lists

Local	Distributed	Common
GALR1	NR2C2	ST8SIA4
NR2C1	HDAC2	HDAC8
ST8SIA4	ST8SIA4	FRY
HDAC8	HDAC8	LONRF3
TFAM	SCN5A	TBL1Y
FRY	NELL1	NR2F1
LOC728734	FRY	NTNG1
TTC21B	ACAP3	EPHA3
SPTBN4	SEC61B	ABCG1
CAMK2B	LONRF3	AUTS2
LONRF3	CABLES1	CAMK1
TBL1Y	TBL1X	SEMA5B
NR2F1	TBL1Y	DLGAP1
ADCY9	ADCY8	IFT20
KLHL3	DEPDC1B	AKAP10
TTC39C	TTC30B	UNC13B
HIST1H1D	NR2F2	PPFIA4
NTNG1	NR2F1	DSP
IDE	MED4	LOC651856
ABCA9	LOC399815	PHTF2
OPRD1	PTPLB	FAXC
EPHA3	PTPN3	KIAA0753
ZADH2	BUB1B	HEATR2
IRF6	PPAPR5	KIAA1324
ST3GAL6	PTPRB	FP588
DNASE1L2	LOC401533	BCL11A
ABCG1	KIAA1919	UBE2D1
RASIP1	HIST1H1A	LIN7A
ABCD2	LRRTM1	CEP57
ABCC1	NTNG1	SUV39H2
RBM15	TTYH3	PPL
ATG16L1	EPHA3	OLFML2A
ATP8B2	EPHA5	CUZD1
NAALAD2	MGC2889	GVINP1
GPR89C	PATE2	ZNF599
DTWD2	C21orf45	NPTN
MIAT	SLC22A10	PPARD

AUTS2	PLXND1	AKAP7
HS3ST1	LOC647012	ZNF643
VPS36	MGC2848	CMKLR1
CEBPG	XRCC5	SMPD3
CAMK1	TCF3	MLL5
SEMA5B	SMARCD1	CDKL2
PCDHA5	TRNT1	ZNF711
BID	ABLIM2	SLC22A9
DLGAP1	ABCG1	RNF133
FOXO3	CYB5B	DDX4
FOXP1	PSG9	THAP1
MASP2	PSG4	RASGEF1B
KIAA0528	CD276	SLC44A5
EXTL2	AC114947.2	TIAM1
SDR16C5	PSMG2	ZNF131
LOXHD1	USP10	CDH12
ANKS1A	DTWD1	MET
IFT20	AUTS2	NAA50
AKAP10	CEBPD	ZDHHC18
TRPC3	C11orf82	NLGN1
UNC13C	TANK	KRTCAP3
UNC13B	UBR7	KIAA1462
SH3BP5L	CAMK1	CCDC88B
POTED	SEMA5B	LRRK2
FKBP7	MAP3K12	COBLL1
ARSG	DLGAP1	ANAPC4
PPFIA4	BMX	PCDH11Y
DSP	FOXK2	PCDH11X
LOC651856	SAFB	SLC25A27
AC110760.2	MINPP1	VAV3
PHTF2	CXorf56	PAPOLB
AFAP1-AS1	ODF2L	SOCS2
C14orf104	BMP2K	SFRS14
FAXC	SHANK3	UXS1
ATP13A3	LOC729983	VCX2
KIAA0753	IFT20	JAK1
PLCG1	AKAP10	FAM20A
HEATR2	CLEC7A	UHRF1BP1
ADNP2	CCNT1	C2orf49
ESRRG	IFT52	SLC17A6

KIAA1324	GALP	MEF2A
FBXO18	RNF11	AC084018.1
RP11-2F9.2	RNF10	PHF14
PDZD8	BZW2	SPTB
LOC200726	BZW1	PRR16
FP588	STAG3L2	C6orf57
PLAGL1	UNC13B	TMEM132E
BCL11A	POTEC	SP9
CEP95	SGIP1	CCDC64
UBE2D1	NLGN4X	ZBTB8A
LIN7A	TAF1A	VCX
CEP57	LINC00328	FAM19A2
SLMAP	TCEAL1	BHLHE22
SRPK1	TCEAL4	CPLX2
SH3RF2	ARL9	ARPP-21
SUV39H2	PRKCDBP	TRAF5
CHRM2	C12orf65	LEPR
PLGLB2	AC105206.1	ANUBL1
LUC7L	PPFIA4	PION
TSHZ1	DSP	RSC1A1
PPL	LOC651856	PHAX
CNTNAP5	PHTF2	ECEL1P2
CENPJ	POMZP3	CRLS1
OLFML2A	WDR67	PLK5P
ACSBG2	C14orf54	LOC100288147
BACH2	GTF2H5	LOC727799
CUZD1	WDR47	LRRC49
KSR2	EAF2	LOC100132261
GVINP1	XYLB	AIM2
AC092143.3	SEC22A	ZCCHC8
ZNF599	ZNRF1	GABRB2
C20orf74	POLE	GABRA6
NOX3	FAXC	KLF13
VSTM2A	CSRNP3	NPY2R
DBC1	KIAA0753	COL13A1
NPTN	HEATR2	PTTG1
PPARD	OR5K4	C5orf24
RAB11FIP2	ZNF781	ODZ2
AKAP7	OGN	ODZ4
MYT1L	ZNF790	PCDHGA3

SLC39A8<br>ZNF643<br>STOX2<br>CMKLR1<br>UMODL1<br>ZNF30<br>GPHN<br>ZNF662<br>SMPD3<br>LAMA4<br>LAMA3<br>ZNF354C<br>MLL5<br>CDKL2<br>HPSE<br>ZNF711<br>SYN3<br>KIAA1797<br>RADIL<br>GPLD1<br>SLC22A9<br>IL7R<br>RNF19B<br>RNF148<br>RNF133<br>SPINT1<br>SAMD12<br>DDX4<br>SPINK2<br>AASDH<br>RAD52<br>THAP1<br>RASGEF1B<br>MRPS5<br>PANK1<br>AP000751.3<br>PFTK1<br>GIT2<br>KCNK12<br>USH2A	ZNF771<br>KIAA1324<br>SNX12<br>FP588<br>FHAD1<br>C4orf49<br>BCL11A<br>PCTK2<br>MRPS17<br>UBE2D1<br>LIN7A<br>ARHGAP10<br>CEP57<br>KIAA1257<br>GNAL<br>TMEM213<br>SUV39H2<br>LOC728024<br>ADAMTS17<br>MOCS3<br>ECM2<br>AC136443.1<br>FOXD4L4<br>C20orf96<br>CISD2<br>PPL<br>AC004053.1<br>OLFML2A<br>ZNF534<br>CUZD1<br>OSBPL10<br>ZNF558<br>GVINP1<br>AC019171.4<br>AC007276.5<br>ZNF599<br>NPTN<br>PPARD<br>AKAP7<br>UBAP2L	METT5D1<br>PCDH7<br>AC125232.1<br>SEPHS2<br>RASA3<br>PDE3A<br>SAMD5<br>CTSC

SLC44A5<br>ZNF445<br>ZNF407<br>SLC12A5<br>ZFAT<br>FGF13<br>TIAM1<br>RYR2<br>ZNF131<br>CDH12<br>SLC35F4<br>MET<br>SLC35E1<br>ATP2B2<br>DYNC2H1<br>NAA50<br>ZDHHC18<br>NLGN1<br>SEPT_6<br>KRTCAP3<br>FSTL1<br>SORCS1<br>DGKK<br>KIAA1462<br>TRIM7<br>OSBP2<br>PAQR9<br>SV2A<br>CCDC88B<br>GIGYF1<br>CACNB4<br>LRRK2<br>COBLL1<br>AC062028.1<br>FAM110A<br>ANAPC4<br>LHFPL4<br>PCDH11Y<br>PCDH11X<br>JARID2	MATR3<br>ZNF639<br>ZNF643<br>ZNF70<br>LOC100134173<br>ZNF18<br>ZNF677<br>AC020663.1<br>ZNF682<br>CMKLR1<br>ZNF780B<br>ZNF670<br>FLVCR2<br>HSPB9<br>RNPC3<br>SMPD3<br>MLF1<br>UBQLN2<br>MLL5<br>CDKL2<br>ZNF711<br>ZNF391<br>ZNF382<br>CUGBP1<br>CWC15<br>DDI2<br>MRPL1<br>ZNF33A<br>MRPL3<br>SLC22A9<br>RIPK1<br>KIAA1772<br>ZNF33B<br>ABCA12<br>RNF133<br>PABPCP5<br>NRN1<br>C6orf182<br>STX17<br>IKZF4	

MSRB3<br>SLC25A27<br>LYPLA1<br>SLC25A37<br>HS6ST2<br>SMN2<br>VAV3<br>RSPO2<br>FAM46C<br>PAPOLB<br>SOCS2<br>SLITRK3<br>SFRS14<br>FAM13B<br>UXS1<br>PCNXL2<br>TRIAP1<br>MTBP<br>TTC28<br>TTC14<br>ELOVL4<br>KRTDAP<br>VCX2<br>AC005277.1<br>HIVEP2<br>DLG5<br>JAK1<br>JAK2<br>FAM20A<br>UHRF1BP1<br>C2orf49<br>EDNRA<br>ENTPD4<br>SLC17A6<br>SETD6<br>SETD7<br>ALDH8A1<br>MEF2A<br>AC084018.1<br>RORB	DDX4<br>METTL2A<br>THAP1<br>RASGEF1B<br>ZNF876P<br>PANK2<br>EXOSC3<br>RAPGEFL1<br>ZFP3<br>SLC44A5<br>ZNF441<br>CDK7<br>CDH2<br>SLC12A8<br>TIAM1<br>SLC9A10<br>KHSRP<br>ZNF131<br>LXN<br>ZNF154<br>ZNF138<br>CDH12<br>MGP<br>MET<br>CNTN5<br>GMDS<br>RAB23<br>RAB12<br>RIMS4<br>NAA50<br>ALG13<br>RAB3B<br>ZDHHC18<br>RAB2A<br>CAP2<br>NLGN1<br>NLGN2<br>SPA17<br>CAV2<br>TMEM97	

RORA<br>CAMK2N2<br>PHF14<br>SPTB<br>MYO19<br>PRR16<br>C6orf57<br>TMEM132E<br>STS<br>SRGAP3<br>ITM2A<br>GRHL2<br>CEP152<br>SLC19A2<br>CEP135<br>PKD2L2<br>C8orf47<br>POU3F2<br>ICA1<br>SP9<br>ZNF280A<br>IFNE<br>LHX9<br>C9orf91<br>VIPR2<br>ITGB3<br>CCDC57<br>CCDC64<br>TMEM86B<br>ZBTB8A<br>CCDC136<br>ZNF385B<br>MXD1<br>C16orf28<br>VCX<br>ZFYVE27<br>UST<br>FAM19A2<br>IGFL4<br>WNT3	KRTCAP3<br>NKIRAS1<br>TMEM154<br>RP11-807H7.1<br>ZNF238<br>C20ORF141<br>TOP1P1<br>TSPAN3<br>KIAA1462<br>KDM2A<br>KIAA1486<br>HNRNPH2<br>ARPC5L<br>CARM1<br>C1QTNF8<br>FAM204A<br>NNT<br>GOT1L1<br>CBX6<br>CBX5<br>CCDC88B<br>INHA<br>LRRC7<br>LRRC6<br>ADNP<br>KNDC1<br>LRRK2<br>SARM1<br>COBLL1<br>FRZB<br>ANAPC4<br>PIAS1<br>OCIAD1<br>CPEB4<br>PCDH11Y<br>PCDH11X<br>SLC25A27<br>C16orf74<br>YIPF4<br>SLC25A16	

LOC100289550<br>BHLHE22<br>CPLX2<br>ARPP-21<br>LEO1<br>TRAF5<br>LEPR<br>ANUBL1<br>PION<br>AC016683.6<br>AC131238.1<br>A2BP1<br>DCBLD2<br>TUBE1<br>KIF15<br>UPP1<br>COL5A1<br>H2BFM<br>KREMEN1<br>CSMD2<br>RSC1A1<br>PHAX<br>INTS8<br>PDIA5<br>RASSF4<br>RASSF6<br>ECEL1P2<br>RBM9<br>TAS2R14<br>IGF1R<br>TAS2R31<br>CRLS1<br>PLK5P<br>CKMT1B<br>LOC100288147<br>SGCG<br>SPSB2<br>LOC727799<br>LRRC49<br>LOC100132261	LSM14B<br>CCBL2<br>VAV3<br>MAMLD1<br>FAM40B<br>ADD2<br>ENY2<br>FAM129A<br>PAPOLB<br>PAPOLG<br>SLC15A3<br>SOCS2<br>SFRS14<br>ASAH2<br>PPP3CB<br>UXS1<br>CHGB<br>BDKRB2<br>CYFIP2<br>PRKCI<br>UBE2W<br>KCNJ9<br>C3orf33<br>AMELX<br>KCND3<br>VCX2<br>JAK1<br>FAM20A<br>UHRF1BP1<br>C2orf56<br>C2orf49<br>ZCCHC10<br>ZCCHC14<br>SPEG<br>ELK4<br>WDR5<br>MSL1<br>SLC17A6<br>MEF2A<br>AC084018.1	

AIM2<br>ZCCHC8<br>TIFA<br>GABRB2<br>GABRA6<br>DGCR8<br>CYTH3<br>PSPC1<br>KLF13<br>PENK<br>LAG3<br>NPY2R<br>SLC38A11<br>C15orf41<br>COL13A1<br>PTTG2<br>PTTG1<br>C5orf24<br>ODZ2<br>ODZ4<br>ZMYM4<br>PCDHGA3<br>LEPREL2<br>PIK3CA<br>LPCAT4<br>METT5D1<br>PHACTR1<br>ASAH2C<br>PCDH7<br>TPTE2P6<br>AC125232.1<br>EYA3<br>CUX1<br>LOC100133686<br>C19orf46<br>SEPHS2<br>FRAT2<br>RASA3<br>PDE3A<br>C1orf172	SCYL2<br>ASCC3<br>CAMK2N1<br>FAM151B<br>GTSF1<br>FAM153B<br>FAM153A<br>PHF14<br>SPTB<br>PRR16<br>PRR12<br>RRN3P3<br>MYO7A<br>C6orf57<br>ERC2<br>KRT40<br>ITIH4<br>CD3EAP<br>FLJ27352<br>TMEM132E<br>RPS7<br>FAM161B<br>ERLIN1<br>LRRIQ3<br>TBC1D9<br>ARID1A<br>APOC1<br>TEF<br>INPP4B<br>LOC100287737<br>PRRT2<br>SKI<br>RSPH10B<br>KALRN<br>SNAP91<br>C8orf38<br>SRC<br>LOC100144595<br>SP3<br>SP9	

RLBP1L1<br>SAMD5<br>DNAJC5G<br>CPNE4<br>CTSC<br>CCDC144NL	MYH2<br>MECP2<br>ZMYND11<br>PPP1R2P3<br>CMBL<br>C9orf41<br>GRM7<br>CCDC64<br>CCDC65<br>CCDC72<br>ZBTB8A<br>CCDC99<br>KANK2<br>VCX<br>CCDC125<br>MTHFD2L<br>RCAN1<br>SNED1<br>STAC<br>N4BP2L1<br>MRPL50<br>SLAMF6<br>DENND1A<br>WASF1<br>FREQ<br>ROD1<br>ZFAND6<br>ZFAND5<br>ZFAND1<br>ATAD5<br>TPM2<br>FAM19A2<br>GNA11<br>THSD1<br>SSBP3<br>SSBP2<br>SUDS3<br>RBP3<br>FAM196A<br>FCGR2B	

	BHLHE22<br>MAPKBP1<br>CPLX2<br>NPTX1<br>ARPP-21<br>IFNA5<br>CCDC109A<br>PKNOX1<br>TRAF5<br>LEPR<br>GTF3A<br>ANUBL1<br>PION<br>GTF2B<br>PAICS<br>AC004863.6<br>LINGO2<br>DCBLD1<br>C7orf57<br>KIF2B<br>C7orf64<br>SCRN1<br>PI15<br>LOC100288237<br>PHF8<br>RSC1A1<br>PHAX<br>TADA1L<br>PDIA3<br>RASSF7<br>TLE1<br>TADA2L<br>ECEL1P2<br>RBM4<br>RBM6<br>TNFAIP3<br>NDUFS4<br>ANO6<br>CRLS1<br>FAM182B	

	PLK5P<br>LOC100288147<br>ACIN1<br>LRFN5<br>FAM185A<br>LDB1<br>BRAP<br>COL14A1<br>SPANXN3<br>LOC727799<br>PIK3C2A<br>AF205589.3<br>AC117392.3<br>LRRC49<br>OTUD1<br>LOC100132261<br>AIM2<br>ZCCHC8<br>CADM3<br>BROX<br>ST6GAL2<br>ABR<br>TMSB15B<br>LRRC37B<br>GABRB2<br>TRIM43<br>GABRA6<br>TRIM55<br>HMGB1P1<br>APC<br>RPAP2<br>GTF2IP1<br>KLF13<br>NPY2R<br>PES1<br>UBLCP1<br>BGLAP<br>COL13A1<br>C15orf33<br>PTTG1	

	AC068491.1<br>FCRL6<br>C5orf24<br>ODZ2<br>ODZ4<br>KRTAP4-8<br>PCDHGA3<br>PCDHGB6<br>C5orf42<br>METT5D1<br>DNAJC5<br>PCDH7<br>LOC100129361<br>AC125232.1<br>EXT1<br>CUX2<br>C19orf44<br>C19orf45<br>C19orf22<br>SEPHS2<br>REXO1<br>RASA3<br>RASA2<br>PDE3A<br>PDE3B<br>PDE5A<br>C1orf135<br>PDE7B<br>CACNA1C<br>MBNL1<br>ATPAF1-AS1<br>DAB2IP<br>DNAJC14<br>SAMD5<br>SAMD9<br>PBX2<br>NRXN2<br>NRXN1<br>CTSC	
